# Direct surface analysis mass spectrometry uncovers the vertical distribution of cuticle-associated metabolites in plants†

**DOI:** 10.1039/d2ra07166e

**Published:** 2023-03-14

**Authors:** Siriel Saladin, Sara D'Aronco, Gwyneth Ingram, Chiara Giorio

**Affiliations:** a Yusuf Hamied Department of Chemistry, University of Cambridge Lensfield Road Cambridge CB2 1EW UK; b Laboratoire Reproduction et Développement des Plantes, ENS de Lyon, CNRS, INRAE, UCBL F-69342 Lyon France

## Abstract

The plant cuticle covers the plant's entire aerial surface and acts as the outermost protective layer. Despite being crucial for the survival of plants, surprisingly little is known about its biosynthesis. Conventional analytical techniques are limited to the isolation and depolymerization of the polyester cutin, which forms the cuticular scaffold. Although this approach allows the elucidation of incorporated cutin monomers, it neglects unincorporated metabolites participating in cutin polymerization. The feasibility of a novel approach is tested for *in situ* analysis of unpolymerized cuticular metabolites to enhance the understanding of cuticle biology. Intact cotyledons of *Brassica napus* and *Arabidopsis thaliana* seedlings are immersed in organic solvents for 60 seconds. Extracts are analyzed using high-resolution direct infusion mass spectrometry. A variety of different diffusion routes of plant metabolites across the cuticle are discussed. The results reveal different feasibilities depending on the research question and cuticle permeabilities in combination with the analyte's polarity. Especially hydrophilic analytes are expected to be co-located in the cell wall beneath the cuticle causing systematic interferences when comparing plants with different cuticle permeabilities. These interferences limit data interpretation to qualitative rather than quantitative comparison. In contrast, quantitative data evaluation is facilitated when analyzing cuticle-specific metabolites or plants with similar cuticle permeabilities.

## Abstract 2

In contrast to conventional analytical methods, direct surface analysis enables the investigation of free cuticular metabolites in plants.

## Graphical abstract

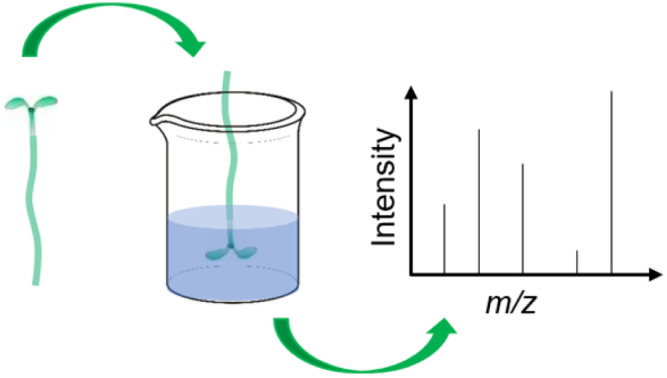

## Introduction

The emergence of land plants around 500 million years ago represents one of the most drastic events in Earth's history.^1^ There were far-reaching geological, climatic and biological consequences. The conquest of dry land by plants led to the formation of soil and new habitats, as well as an increase in atmospheric oxygen, strengthening the ozone layer and creating the basis for further development of complex life.^2^

Although the newly gained habitat offered great advantages, such as easier access to carbon dioxide for photosynthesis, terrestrialization also brought novel challenges that had to be overcome.^2^ The absence of water led to more exposure to ultraviolet radiation, while the presence of atmospheric oxygen, generated in part by the plants themselves, resulted in increased oxidative stress. Plants needed to develop more rigid structures due to the lack of buoyancy and the increased effects of gravitation. Life on land was furthermore accompanied by more extreme and fluctuating temperatures. At the same time, the first land plants were exposed to new pests such as microorganisms or, later, insects. Finally, terrestrial life goes hand in hand with a limited availability of water: plants had to develop the ability to store water, to prevent evaporation and to cope with dry periods.^2^

Facing terrestrial constraints, the plant surface played a crucial role as the interface between the organism and its environment. Not surprisingly, it was precisely at the plant surface where major adaptations took place leading to the formation of the plant cuticle. The cuticle is a waxy barrier covering the plant's entire aerial surface and protecting it from ultraviolet radiation, oxidative stress, extreme temperatures, pests and desiccation. Besides its protective function, the cuticle plays a critical developmental role, such as by preventing fusion of growing organs.^3,4^ These important functions are thought to have been critical for the evolution of higher plants.

**Fig. 1:**
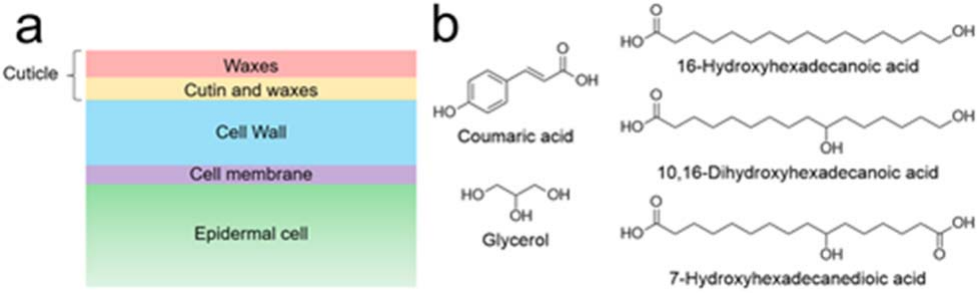
(a) Schematic cross section of a plant leaf surface. The transition between both cuticular compartments (red and orange) is a continuum rather than a well-defined line. (b) Examples of typical cutin monomers.^8^

The plant cuticle is not a homogeneous matrix. Instead, it is a complex, multi-layered structure made up of different interconnected components (Fig. 1a). It can be thinner than 50 nm, as in leaves of *Arabidopsis thaliana*,^5^ or thicker than 200 μm, as in the cactus *Ariocarpus fissuratus*.^6^ Most cuticles have a hydrophobic surface made of waxes, mostly very-long-chain aliphatic compounds (16 to 36 carbon atoms) and their oxygenated derivatives, including alcohols, aldehydes, ketones, fatty acids and esters.^7^ These waxes can be amorphous as well as crystalline and they occur both inside the cuticle and on its surface.^8^ The lipid layer causes poor surface wettability – water droplets roll off and microorganisms find it more difficult to invade the plant. In addition to the self-cleaning effect, the wax leads to a strongly reduced permeability – the exchange of water is restricted, and the plant is protected from desiccation.^8^

Underneath the superficial wax layer is a framework of cutin, dominating the character of the lower cuticle. This highly cross-linked polyester consists of various monomers (Fig. 1b). These are mainly oxygenated fatty acids with 16 or 18 carbon atoms and differ from one plant to another.^8^ Furthermore, other monomers such as dicarboxylic acids, unsaturated fatty acids, glycerol and phenylpropanoids can be found in cutin, albeit in lower quantities.^8,9^ Often, terminal and midchain hydroxy groups occur, being responsible for cross-linking.^8^ It is known that fatty acid monomers are synthesized in the chloroplasts of epidermal cells,^10^ but it is unclear how these hydrophobic molecules traverse the hydrophilic cell wall to reach the polymerization site. Cutin is believed to be covalently bound to polysaccharides of the underlying cell wall.^11–13^ However, the extent of the interaction between cutin and the cell wall remains poorly understood, as does the polymerization mechanism and microstructure of cutin.

Despite being crucial for the survival of plants, surprisingly little is known about the biosynthesis of plant cuticle. Many of the chemical findings about cutin polymerization originate from decades of research, whereby the already fully developed cuticle is isolated and chemically decomposed into its components.^14–16^ According to the monomers found, an attempt is then made to reconstruct the structure and synthesis of cutin. However, this “isolation and depolymerization” based approach has two major limitations. Firstly, only molecules incorporated into the polymer can be identified. Other metabolites involved in polymerization without being incorporated into the polymer are lost during polymer extraction. Secondly, this approach mainly examines mature plants in which cuticle development has already been completed and metabolic activity has presumably ceased, resulting in a loss of information.

Direct surface analysis of plant leaves coupled to high-resolution mass spectrometry, as described by Giorio *et al.*,^17,18^ appears to be a promising alternative strategy. Placement of a drop of solvent on the plant cuticle and subsequent aspiration of the same drop allows *in situ* analysis of cuticular metabolites (typical extraction times of ≤60 seconds). Free metabolites that accumulate in the cuticle during cuticle development and cutin polymerization are thus potentially accessible for research and not washed away. Spatial resolution is inherent in the data obtained, which makes *in situ* analysis fundamentally different from conventional chemical cuticle analyses.

However, direct surface analysis as performed by Giorio *et al.*^17,18^ faces constraints. Firstly, the technique requires a flat plant surface large enough to accommodate a drop of solvent (3 μL), which is typically not found in roots, stems or the seed leaves (cotyledons) of young seedlings. Secondly, it is not clear to what extent the extracted metabolites derive from the cuticle itself or from the matrix beneath the surface. Thus, data interpretation becomes complex.

In this study, cotyledons of plants were immersed in extraction solvent to overcome the size- and shape-dependent limitations described above. Several experiments with different solvents, plants and plant organs were conducted to shed light on the diffusion pathways across the cuticle, to understand the distribution of cuticle-associated metabolites and to assess whether direct surface analysis of cuticular metabolites can be used as a tool for both metabolomic studies and to understand plant surface biogenesis.

## Experiments

### Plant material

Wild type *Brassica napus* was purchased as commercial seedlings in an organic substrate from Tesco UK. Seedlings had two fully developed cotyledons but no true leaves. Col-0 and *gso1 gso2* seeds of *Arabidopsis thaliana* were provided by Dr Gwyneth Ingram (Laboratoire Reproduction et Développement des Plantes, ENS de Lyon) according to György P. Rédei^19^ and Tsuwamoto *et al.*^20^ Seeds were sterilized for 15 min in 70% ethanol (Sigma-Aldrich) with 0.05% Triton™ X-100 (Sigma-Aldrich), rinsed three times with ≥99.8% ethanol (Sigma-Aldrich) and air dried on filter paper. New filter papers were taken, soaked in tap water, allowed to drain for approximately two seconds and placed in Petri dishes. The seeds were sprinkled onto the wet filter papers before closing and sealing the dishes. After 72 hours of stratification at 4 °C in darkness, the Petri dishes were placed next to the window for six hours and exposed to daylight to trigger germination (room temperature, no direct sunlight). The Petri dishes were then stored in darkness at room temperature for five days. Seedlings were etiolated, had yellowish cotyledons and colorless hypocotyls. Plant material was incinerated after use.

### Conditions prior to extraction

*A. thaliana* seedlings were kept in darkness at room temperature before analysis (Petri dish atmosphere). *B. napus* seedlings were conditioned in the laboratory at room temperature and ambient atmosphere for at least one hour prior to sample extraction (normal daylight) if not otherwise stated. In some cases, the *B. napus* seedlings were placed for one hour in desiccators with a saturated solution of sodium chloride leading to 75% relative humidity or sodium hydroxide leading to 15% relative humidity. Desiccators were either wrapped in aluminum foil (darkness) or placed next to the window (light) without direct sunlight. Seedlings in the desiccators were either fully exposed to air (no water) or placed in tap water (water). The roots were cut from the hypocotyls using a sharp scalpel before placing the plants in the desiccators. During the chemical trace analysis, the plants were kept at room temperature and ambient atmosphere next to the window without direct sunlight (roots of seedlings were removed by cutting the hypocotyl with a sharp scalpel).

### Sample extraction

Roots or cotyledons of *A. thaliana* seedlings were extracted for 60 s by immersion in 50 μL of acetonitrile(ACN)/water 90 : 10 containing 0.1% formic acid (FA) as modifier and 0.1 mg L^−1^ 4-chlorobenzoic acid (Sigma-Aldrich) as internal standard (IS) if not otherwise stated (one individual seedling per extraction). 0.5 mL of the same solvent was used for extraction of *B. napus* cotyledons (60 s extraction time). All plants were immersed in the solvent using tweezers in such a way that only the roots or cotyledons were in contact with the solvent. The other parts of the plants as well as the tweezers themselves were not immersed. No sectional cuts were made to avoid contamination by damaged tissues. The samples from the subsections ‘Polar metabolites on cuticle surface’ and ‘Delipidation’ were extracted with different solvents. To eliminate the effect of different ionization efficiencies, these extracts were diluted prior to analysis into a final solution of ACN/water 50 : 50 and ACN/CHCl_3_/water 49 : 49 : 2, respectively. Further details on the dilution of the extracts can be found in Tables S1 and S2.† Water (Fisher Scientific), ACN (Fisher Scientific), chloroform (CHCl_3_, Sigma-Aldrich) and FA (Thermo Scientific) were of LC/MS grade.

### Mass spectrometry

Extracts were analyzed with a chip-based nanoESI source (Triversa NanoMate®, Advion) in negative ion mode with a voltage of −1.40 kV, gas pressure of 0.8 psi and a HD ESI A chip (nozzles with 5.5 μm internal diameter, Advion) coupled to a LTQ Velos Orbitrap (Thermo Fisher Scientific) mass spectrometer (MS) with a resolution of 100 000 at *m*/*z* 400 and a typical mass accuracy within ±2 ppm. The instrument was routinely calibrated according to the instructions of the manufacturer using a Pierce™ LTQ ESI Positive Ion Calibration Solution and a Pierce™ ESI Negative Ion Calibration Solution (Thermo Fisher Scientific). Transfer capillary temperature was set to 210 °C. Two different *m*/*z* ranges were acquired for each sample: 80–600 and 150–1000. MS/MS analysis was done for the five most intense signals with a collision-induced dissociation (CID) energy of 30 (normalized collision energy). A minimum of 10 scan routines was acquired per sample (*ca.* 1 minute of acquisition). In general, data were acquired for more than 2 minutes. The signal at *m*/*z* 311.16864 (C_17_H_28_SO_3_, sulphophenyl-undecane) is a common contaminant and was used as a lock mass. Fig. S1 (ESI†) shows typical mass spectra.

### Data processing

Results were processed with Xcalibur™ (Thermo Fisher Scientific) and a Mathematica 10 (Wolfram Research) code written in-house. The code runs noise elimination, corrects the mass drift using up to 21 common contaminants with known masses and removes shoulder ions. Furthermore, blank subtraction was done by deleting all signals with a sample to blank ratio of less than 10. The code assigned one or more chemical formulas to each signal, considering a maximum allowed mass error of ±5 ppm and the following parameters: 1 ≤ C ≤ 100, 1 ≤ H ≤ 200, N ≤ 5, O ≤ 50, S ≤ 2, O/C ≤ 2, 0.3 ≤ H/C ≤ 2.5, N/C ≤ 1.3 and S/C ≤ 0.3. Multiple assignments for a single peak were allowed. Further details on data processing are described in the publication of Zielinski *et al.*^21^ The figures in this work show a.u. (arbitrary units) raw values on a logarithmic scale and standard deviations as error bars.

## Results and discussion

In this work, surface metabolites from *A. thaliana* and *B. napus* (rapeseed) cotyledons were extracted with mixtures of water, ACN and CHCl_3_. To avoid misleading conclusions, it is essential to understand the diffusion pathways through which the metabolites could reach the surface and be dissolved by the solvent during extraction. The word “surface” is potentially misleading. The interface of the plant outer cell layer (epidermis) with its environment is not a homogeneous, two-dimensional layer. Instead, it is a heterogeneous and three-dimensional structure consisting of various molecules and cell types such as pavement cells or stomata.^22^ These are covered in a highly heterogeneous multi-layered zone of extracellular matrix. Where does the “surface” begin and where does it end? Where do extracted molecules come from when surface extraction is performed? The answers to these questions are far from trivial.

### Polar metabolites on cuticle surface

**Fig. 2:**
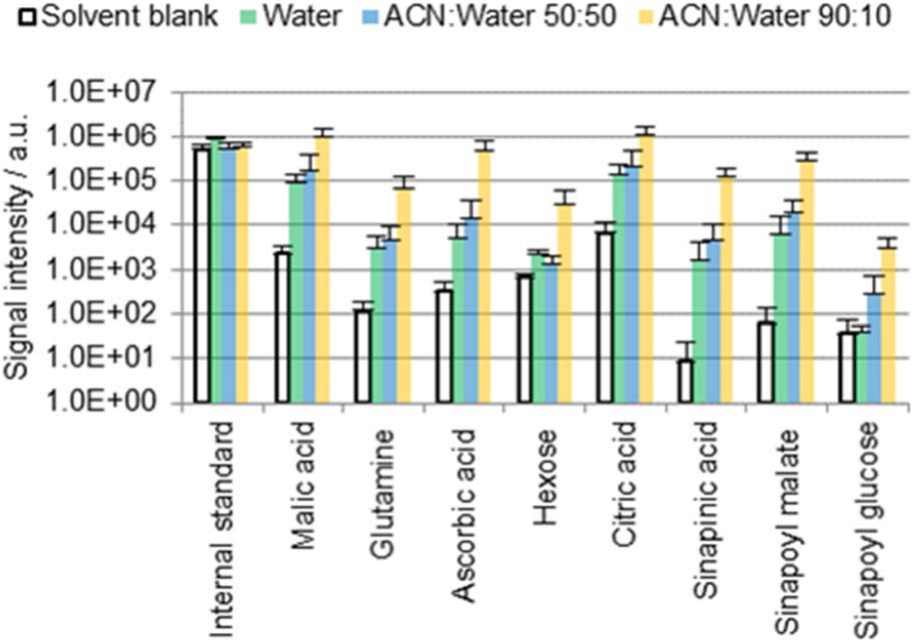
Results of the direct surface analysis MS of the *B. napus* cotyledons immersed in different solvents containing 0.1% FA. Averages of three replicates with individual seedlings are shown.

One possible location for cuticular metabolites is the outermost surface of the cuticle, which in many (but not all) cases contains epicuticular waxes. Polar metabolites are not intuitively expected to be found there given the nonpolar character of the epicuticular waxes. This was tested empirically by immersing cotyledons of *B. napus* seedlings for 60 s in 0.5 mL of three different extraction solvents: water, ACN/water 50 : 50, or ACN/water 90 : 10 (Fig. 2). Solvents contained 0.1 mg L^−1^ IS and 0.1% FA.

Fig. 2 shows a selection of the most abundant metabolites that were detected. Table S3 (ESI†) provides an overview of selected chemical formulas that were (not) detected. Phenylpropanoids are of particular interest as they may play a crucial role in the structure of the cutin polymer, for example as a potential covalent linker between cutin and the cell wall.^23,24^ However, with the exception of sinapinic acid and its conjugates, no other phenylpropanoids were detected with a moderate intensity (signal-to-noise ratio higher than 100). Sinapinic acid is a characteristic phenylpropanoid in plants of the *Brassicaceae* family^25,26^ such as *B. napus*,^27^*A. thaliana*^28^ or the eponymous *Sinapis alba*^29^ (white mustard). The presence of malic acid,^30^ glutamine,^31^ ascorbic acid,^30^ hexose,^32^ citric acid,^30^ sinapinic acid,^27^ sinapoyl malate^28^ and sinapoyl glucose^27,28^ in *Brassicaceae* leaves has been reported before. The same applies to malic acid, glutamine, ascorbic acid, hexose and citric acid on petal surfaces of *Hibiscus trionum*.^17^

Many of the detected metabolites (hexose, organic acids) are more soluble in water than in ACN.^33^ Nevertheless, signals were found to be up to two orders of magnitude more intense when extractions were carried out with an ACN/water solvent mixture rather than with 100% water (Fig. 2). If the molecules were localized exclusively on the outermost cuticle surface, more intense signals would be expected when extracting without ACN due to a greater solubility in water.^33^ Consequently, it is suggested for the ACN/water 90 : 10 data that a large fraction of the detected molecules may originate from the inner part of the cuticle or the underlying epidermal extracellular matrix rather than from the cuticle surface.

A fraction of these metabolites was however extracted in 100% water, suggesting that these polar metabolites may be present also on the cuticle surface (as possible residues of guttation, or as residues from the endosperm that surrounds the cotyledons prior to their germination). The contribution of the superficial fraction to the detected metabolite pools appears to be minor compared with that from the intracuticular or subcuticular matrix given the large difference between the results obtained using water and ACN/water 90 : 10 (Fig. 2).

Unexpectedly, the difference between the results obtained with water and ACN/water 50 : 50 is lower than the difference between ACN/water 50 : 50 and 90 : 10. The organic solvent seems to support or enable a diffusion pathway which is not, or only weakly, promoted by water. It seems that the diffusion process may not depend on the solvent composition in a linear fashion. One possible explanation is that the presence of the solvent may promote stomatal or hydathodal infiltration.

### Stomatal and hydathodal infiltration

Stomata and hydathodes are pores found on the surfaces of many aerial plant organs. Whilst stomata regulate the exchange of gas, hydathodes secrete water enriched with certain solutes (guttation). Both types of pores are regulated by abiotic stimuli such as light and water.^34,35^ The more water and light present, the more likely the pores will open.^34^

**Fig. 3:**
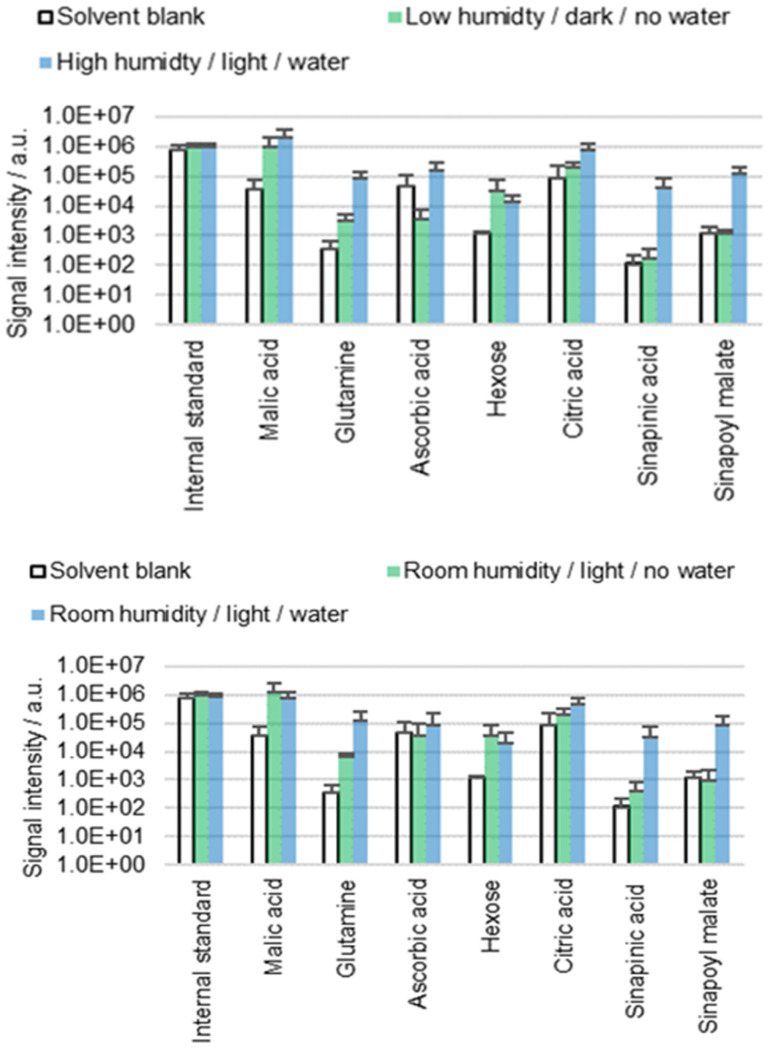
Results of the direct surface analysis MS of the *B. napus* cotyledons. Seedlings were kept under different conditions for one hour prior to extraction by immersion in ACN/water 90 : 10 containing 0.1% FA. Averages of three replicates with individual seedlings are shown.

*B. napus* cotyledons were extracted for 60 s with ACN/water 90 : 10 containing 0.1% FA. Seedlings were exposed for one hour to different humidity levels (low, high or ambient) and different light conditions (darkness or daylight) prior to extraction. The hypocotyls were either placed in tap water (water) or exposed to air (no water) to investigate whether these conditions impact the results of surface analysis (Fig. 3). See the subsection ‘Conditions prior to extraction’ in the Materials and methods section for more details.

The results (Fig. 3) suggest that diffusion behavior is affected by the conditions prior to extraction, especially in the case of the two phenylpropanoids sinapinic acid and sinapoyl malate. Interestingly, the factors light and humidity did not show an influence as significant as the factor water. It is unclear to what extent this result is related to stomata or hydathodes since we were unable to measure aperture prior to extraction. However, the obtained results suggest the presence of at least one diffusion pathway, which is facilitated by the placement of the hypocotyl in water. The water dependence leads to the hypothesis that this diffusion pathway is used by molecules from a subcuticular space, which goes hand in hand with the hypothesis of stomatal or hydathodal infiltration. On the other hand, placing the plants in water caused turgor pressure to increase, which could be observed unambiguously. It is conceivable that the increased turgor pressure causes physiological changes that lead to tension in the cuticle and altered cuticle permeability. However, it is unclear whether the observed correlation between water and extraction behavior is attributable to stomatal/hydathodal infiltration, turgor pressure-dependent mechanical surface alterations, or to another mechanism.

The idea that apoplastic molecules diffuse through stomata or hydathodes during extraction is consistent with literature, although most of the published studies address foliar absorption: diffusion of external material such as pesticides or pathogens into the plant's interior.^36^ However, if molecules diffuse through stomata in one direction, then diffusion in the other direction seems to be equally plausible. It was reported that stomatal infiltration depends on the morphology of the stomata and on the surface tension of the applied fluid.^37,38^ Stomata are generally designed to prevent water from entering even when they are open, due to surface tension. In contrast, organic solvents with low surface tension or water with surfactants are known to enter stomata,^37^ which is consistent with the idea that stomatal infiltration may contribute to the phenomena described in this work. However, the results do not indicate that the detected metabolites are extracted exclusively by stomatal or hydathodal infiltration, especially as for some molecules (malic acid, hexose or citric acid) no significant difference between the different conditions is seen.

### Delipidation

**Fig. 4:**
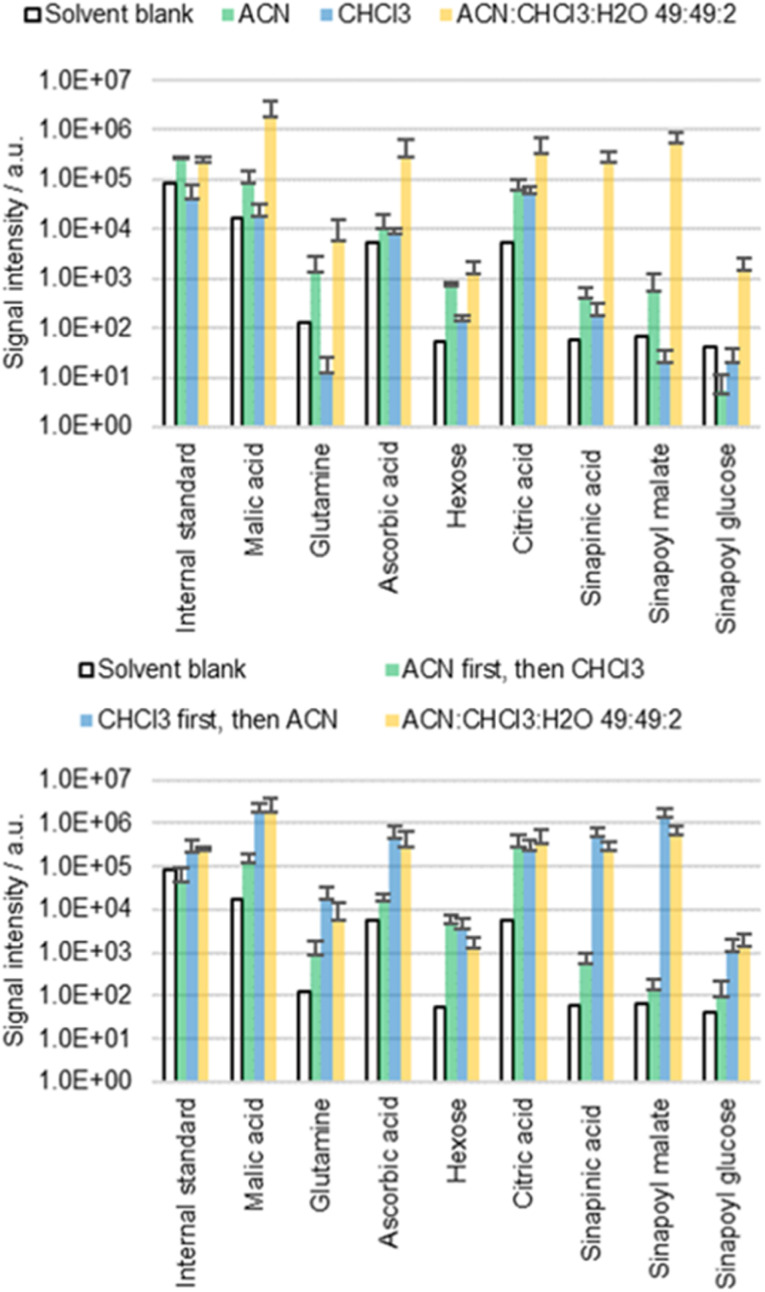
Results of the direct surface analysis MS of *B. napus* cotyledons either immersed for 60 s in ACN, CHCl_3_, or a mixture of both or first immersed for 30 s in ACN (or CHCl_3_) and then 30 s in CHCl_3_ (or ACN respectively). The extract of the first solvent was disposed, while the extract of the second solvent was analyzed with MS. Averages of three replicates with individual seedlings are shown.

The cuticle is not a homogeneous layer, instead it is characterized by a marked transverse heterogeneity with a waxy outer layer representing the major resistance to diffusion.^39^ If the waxy lipid layer on the cuticle is indeed the main barrier preventing diffusion, then CHCl_3_ would be expected to release more metabolites than ACN. CHCl_3_ is well known to dissolve epicuticular waxes providing access to the underlying metabolites.^40–42^ In order to understand the diffusion behavior, cotyledons of *B. napus* were extracted with ACN, CHCl_3_ and an ACN/CHCl_3_/water 49 : 49 : 2 mixture (top part of Fig. 4). These solvents contained 0.002% FA and 0.1 mg L^−1^ IS.

The results obtained with pure CHCl_3_ were not as conclusive as initially expected. Although intense signals could be detected with ACN/CHCl_3_/water 49 : 49 : 2, the extractions with ACN or CHCl_3_ alone resulted in extracts with low concentrations. It was speculated that although CHCl_3_ can dissolve cuticular waxes, it is unsuitable for extraction of polar metabolites in the apoplast due to the lack of polarity. Consequently, the combination of both ACN and CHCl_3_ might be needed to solubilize first the waxes and second the analytes that become accessible.

This hypothesis was tested empirically by extracting the same sample with two consecutive extractions (bottom part of Fig. 4). First, the cotyledons were immersed for 30 s in 0.5 mL of ACN or CHCl_3_, followed by a second 30 s extraction in 0.5 mL of CHCl_3_ or ACN, respectively. The extracts of the first run were disposed, while the extracts of the second run were analyzed. The results were compared to cotyledons extracted twice for 30 s in 0.5 mL of ACN/CHCl_3_/water 49 : 49 : 2. These solvents again contained 0.002% FA and 0.1 mg L^−1^ IS. The extraction efficiency was significantly different depending on the sequence in which extraction was carried out. Extracting first with ACN and then with CHCl_3_ resulted in low metabolite concentrations. However, the opposite order led to high concentrations, confirming the predicted roles of the two solvents.

The dual role (delipidation and extraction) of the ACN/CHCl_3_/water 49 : 49 : 2 solvent mixture reveals insights into the extraction behavior of the detected molecules. Extraction associated with delipidation suggests a direct diffusion pathway from the cuticle into the solvent. In contrast, the solvent ACN/water is not known to delipidate cuticles. Direct diffusion across the cuticle is expected to be less relevant when extracting with this solvent.

### Cuticle permeability

**Fig. 5:**
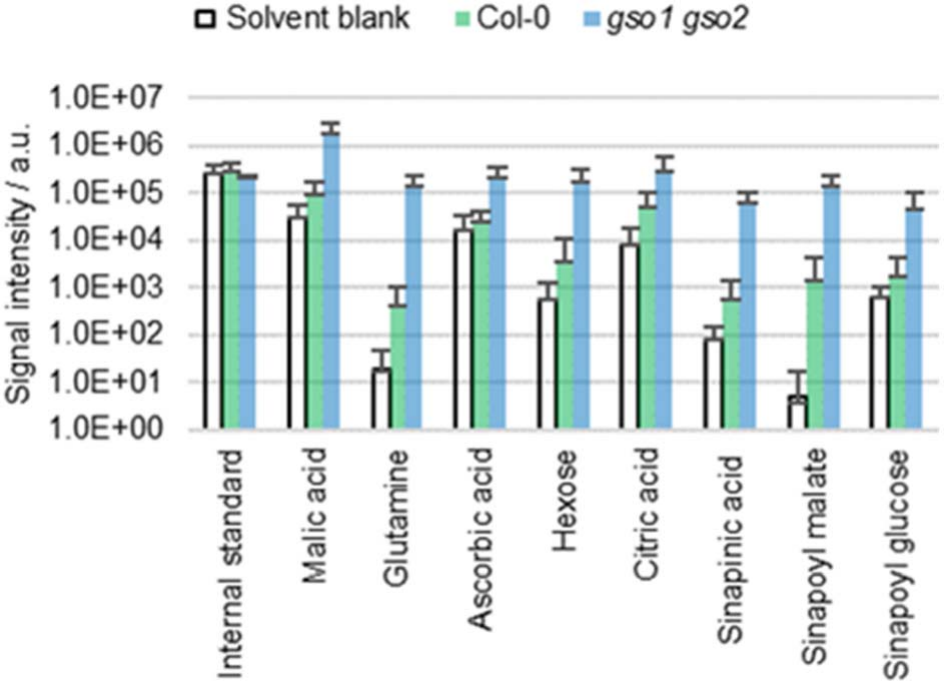
Results of the direct surface analysis MS of the cotyledons of *A. thaliana* wild type (Col-0) and *gso1 gso2* mutants immersed in ACN/water 90 : 10 containing 0.1% FA. Averages of five replicates with individual seedlings are shown.

Plants such as the *gso1 gso2* mutant of *A. thaliana* are known to have an altered cuticle integrity compared to the wild type.^43^ Given the deficient cuticle, extracts of *gso1 gso2* mutants are expected to be more concentrated than those of the wild type, which was tested empirically by extracting cotyledons of both genotypes with ACN/water 90 : 10 (Fig. 5). The extracts from the mutant are significantly more concentrated compared to those of the wild type, which is not surprising given the increased cuticle permeability.^43^ Thus, extraction efficiency seems to depend on cuticle permeability.

### Analysis of roots

It is still unclear whether the detected metabolites derive from the cuticle or the underlying matrix (for example the cell wall). The presence of the cuticle is most unfavorable for the investigation of free metabolites in the cell wall due to the cuticle's role as a bidirectional barrier. However, in roots, only the freshly germinated/emerged root caps are covered with a (highly permeable) cuticle,^44^ while the main parts are cuticle-free to facilitate water uptake. The lack of cuticle in roots simplifies direct surface analysis of the cell wall.

**Fig. 6:**
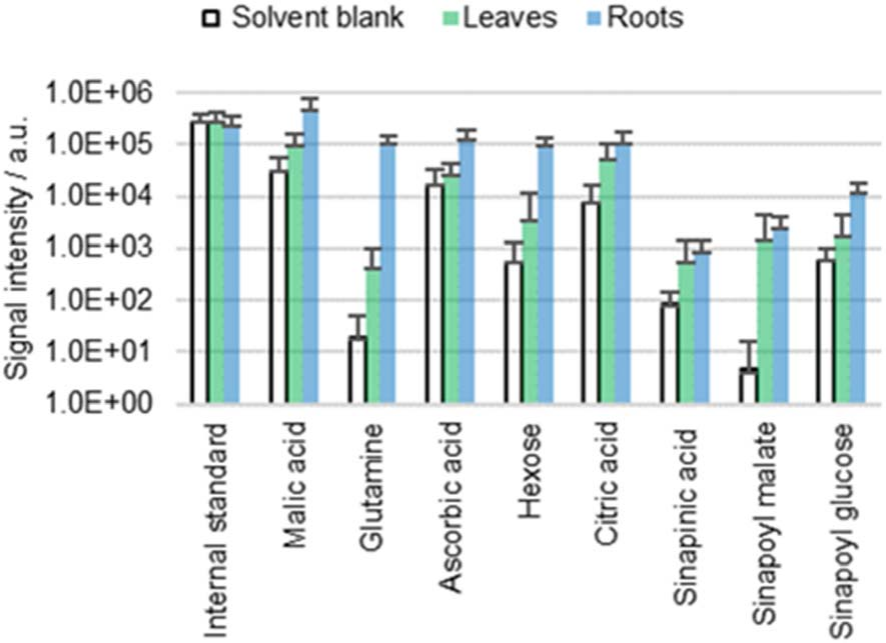
Results of the direct surface analysis MS of the cotyledons (leaves) and roots of wild type (Col-0) *A. thaliana* immersed in ACN/water 90 : 10 containing 0.1% FA. Averages of five replicates with individual seedlings are shown.

Chemical composition of free metabolites in both cotyledons and roots of wild type *A. thaliana* was compared using ACN/water 90 : 10 with 0.1% FA and 0.1 mg L^−1^ IS as a solvent (Fig. 6). Comparison reveals no significant qualitative difference in the metabolic profile of the cotyledons and the roots. Quantitatively, sinapinic acid and its conjugates appear to be more cuticle-specific than for example glutamine, for which detection rates differ significantly between roots and cotyledons. Furthermore, the roots appear to have a higher permeability, which is not surprising given the lack of cuticle.

This result suggests that similar molecules can be found throughout different surface parts of plants. The detection of analytes in the plant's surface is no prove that these metabolites are cuticle-specific.

### Chemical trace analysis

**Fig. 7:**
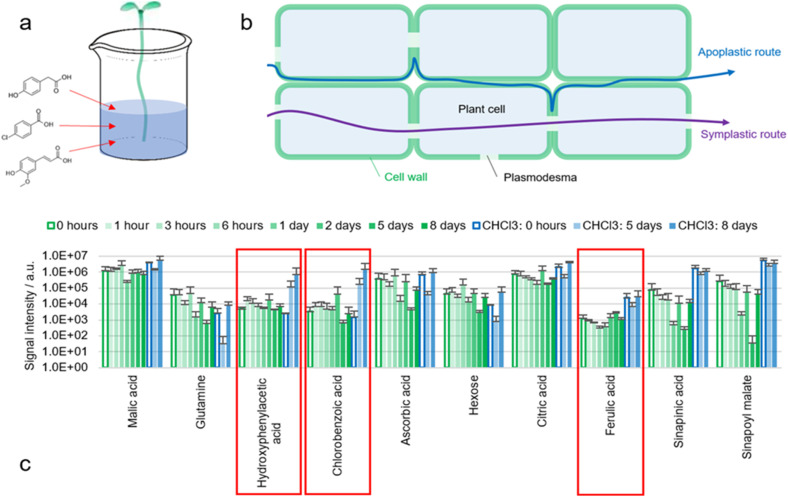
(a) Illustration of the experiments in which hypocotyls of *B. napus* seedlings (no roots) were placed in tap water containing 4-hydroxyphenylacetic acid, 4-chlorobenzoic acid and ferulic acid as chemical tracers (100 mg L^−1^ each). (b) Illustration of apoplastic and symplastic transport routes. (c) Results of the direct surface analysis MS of the cotyledons of *B. napus* seedlings at different timepoints, in which the seedlings were decapitated, and the cotyledons were immersed in ACN/water 90 : 10 containing 0.1% FA. Extracts of three individual seedlings per timepoint were analyzed with MS and compared to seedlings placed in tap water without any marker (0 hours exposure). On day five and eight, the already extracted seedlings were measured again with ACN/CHCl_3_/water 49 : 49 : 2 containing 0.1% FA (blue bars).

A proven procedure in analytical chemistry to understand diffusion pathways is the use of chemical tracers. A known molecule is added to the sample at one location and then recovered at another location (Fig. 7a).

The roots of *B. napus* seedlings were removed by cutting the hypocotyl with a sharp scalpel. The bases of the hypocotyls were submerged in tap water alone, or in an aqueous solution of three tracers: 4-hydroxyphenylacetic acid, 4-chlorobenzoic acid and ferulic acid (100 mg L^−1^ each). Water was absorbed *via* the xylem due to transpirational pull and distribution of the tracers within the plant was expected. An increase of turgor pressure could be observed visibly. 4-Hydroxyphenylacetic acid and 4-chlorobenzoic acid were not only taken up by the plant but also recovered during extraction (Fig. 7c). Contamination seems unlikely as otherwise the markers would have been detected already in the first analysis with ACN/water 90 : 10.

Detection appears to be possible exclusively when the cuticle is delipidated with CHCl_3_. This suggests that the markers reach the epidermis but are retained within the epidermal cell wall by the cuticle. However, since other metabolites such as malic acid are found even without delipidation, it seems likely that either the concentration of absorbed markers was insufficient, or that diffusion pathways with different selectivities are present.

An increase in the ferulic acid signal could not be observed (nor feruloyl malate or feruloyl hexose). It is possible that ferulic acid is metabolized and for example oxidized to sinapinic acid. This is in accordance with the fact that hardly any free ferulic acid is found in the surface of untreated seedlings.

Detection of the two markers (4-hydroxyphenylacetic acid and 4-chlorobenzoic acid) leads to the question of how these molecules are transported within the plant and where they accumulate in the plant surface. Since chlorinated aromatic compounds are not natural metabolites, it seems questionable whether plants possess proteins to actively transport chlorobenzoic acid across cell membranes. Consequently, it seems more likely that 4-chlorobenzoic acid uses the apoplastic instead of the symplastic transport system (Fig. 7b).

Other studies reported the presence of polar metabolites such as malic acid, citric acid and other organic acids in the xylem fluid of several plants.^45,46^ The average concentration of organic acids found in the xylem sap of leaves from several Fagaceae ranged between 1.5 and 2 mM and was about five times lower than the concentration of inorganic cations.^46^ This suggests that organic anions move freely in the plant's cell walls as proposed in this work, although presumably not as freely as inorganic cations. It has been observed that anion mobility in plant cell walls is more constrained than cation mobility.^36,47^ This is probably due to pectic acid, which is incorporated in the cell wall resulting in a slightly negative net charge.^48^

## Conclusions

Understanding the localization of the analytes within the plant surface is essential to assess the feasibility of the novel strategy presented in this work. The results illustrate that particularly polar metabolites such as malic acid, glutamine, ascorbic acid, hexose, citric acid, sinapinic acid and its conjugates are likely distributed throughout the apoplastic network in the examined plants (*B. napus* and *A. thaliana*). The cell walls act as transport pathways, driving diffusion within the plant. It is suggested that polar metabolites are extracted from the cuticle itself as well as from the underlying cell wall when performing direct surface analysis of plant leaves with ACN and water.

Directly above the epidermal cell wall is the lowermost part of the cuticle, which is characterized by cutin. This cuticular layer is called sorption compartment and has empirically shown to possess an increased mobility of polar metabolites.^39,49^ As a consequence, a passive two-way transport of metabolites between the lower cuticle and the epidermal cell wall can be assumed, leading to harmonization of the chemical profiles of the two compartments. In other words, the non-selective extraction of intra- and subcuticular metabolites by itself is not necessarily critical, as the polar metabolites from both compartments may be equally involved in cuticle biology due to the dynamic exchange described above. Metabolites from the epidermal cell wall and metabolites in the cuticle are of equal interest for the study of cuticle biology and there is no implicit necessity to separate intracuticular from subcuticular metabolites depending on the research question. Therefore, the novel strategy presented in this work can be used for metabolomic studies when comparing plants with similar cuticle permeability, when analyzing cuticle-specific analytes or when conducting qualitative measurements.

However, when genotypes with different cuticle permeabilities are compared, different extraction efficiencies inevitably occur, making quantitative data evaluation more complex. It is helpful if nonpolar rather than polar analytes are studied. Nonpolar metabolites such as fatty acids are less likely to be present in the bulk cell wall. Therefore, extraction efficiency of nonpolar analytes may be less dependent on cuticle permeability allowing quantitative comparison between plants with different cuticle integrities. The presence of metabolites in the cell wall can be tested by comparing extracts from leaves with extracts from roots of the same plant.

There is another exception facilitating quantitative comparison between plants with different cuticle integrities. If the genotype with higher cuticle permeability has a lower superficial analyte level, the quantitative results can be conclusive, despite differences in extraction efficiency. The genetically induced decrease in analyte level can be greater than the permeability induced increase in extraction efficiency. Therefore, the latter bias can be neglected with good confidence, if the mutant shows lower analyte levels than the wild type.

Finally, we have demonstrated the value of using non-metabolized tracers like 4-hydroxyphenylacetic acid or 4-chlorobenzoic acid to study cuticle properties. Metabolite concentrations could be expressed as a ratio to the tracer rather than as absolute values to improve the scope of analyses comparing samples with variable cuticular permeabilities. The tracer can be introduced *via* the xylem into the cell walls of both wild type and mutant plants (Fig. 7a).

## Conflicts of interest

There are no conflicts to declare.

## Supplementary Material

RA-013-D2RA07166E-s001
