# Supplementary material for: Direct surface analysis mass spectrometry uncovers the vertical distribution of cuticle-associated metabolites in plants
Source: RSC Adv. 2023 Mar 14;13(13):8487–95. doi: 10.1039/d2ra07166e (PMC10012332; doi:10.1039/d2ra07166e)
Supplement: RA-013-D2RA07166E-s001 [file RA-013-D2RA07166E-s001.pdf]

Electronic Supplementary Material (ESI) for RSC Advances.
This journal is © The Royal Society of Chemistry 2023

Supporting Information for

Direct surface analysis mass spectrometry uncovers the vertical distribution of cuticle-associated metabolites in plants

Siriel Saladin,[a] Sara D'Aronco,[a] Gwyneth Ingram,[b] Chiara Giorio[a]

[a.] *Yusuf Hamied Department of Chemistry, University of Cambridge, Lensfield Road, Cambridge CB2 1EW, United Kingdom*

[b.] *Laboratoire Reproduction et Développement des Plantes, ENS de Lyon, CNRS, INRAE, UCBL, F-69342, Lyon, France*

Table S-1 Different solvents have different ionization efficiencies. Hence, comparison between different extraction solvents is only feasible if the extracts are diluted to a defined ionization solvent prior to infusion into the MS. This was achieved as described in this table for the data of the subsection 'Polar metabolites on cuticle surface' (*B. napus*, Fig. 2). ACN:water 50:50 (0.1 % FA and 0.1 mg L^{-1} IS) was used as a solvent blank.

Extraction solvent	Diluted with	Dilution factor	Ionization solvent
0.5 mL of water (0.1 % FA and 0.1 mg L^{-1} IS)	0.5 mL of ACN (0.1 % FA and 0.1 mg L^{-1} IS)	2	ACN:water 50:50 (0.1 % FA and 0.1 mg L^{-1} IS)
0.5 mL of ACN:water 50:50 (0.1 % FA and 0.1 mg L^{-1} IS)	0.5 mL of ACN:water 50:50 (0.1 % FA and 0.1 mg L^{-1} IS)		
0.5 mL of ACN:water 90:10 (0.1 % FA and 0.1 mg L^{-1} IS)	0.5 mL of ACN:water 10:90 (0.1 % FA and 0.1 mg L^{-1} IS)		

Table S-2 Different solvents have different ionization efficiencies. Hence, comparison between different extraction solvents is only feasible if the extracts are diluted to a defined ionization solvent prior to infusion into the MS. This was achieved as described in this table for the data of the subsection 'Delipidation' (*B. napus*, Fig. 4). ACN:$CHCl_3$:water 49:49:2 (0.002 % FA and 0.1 mg L^{-1} IS) was used as a solvent blank.

Extraction solvent	Diluted with	Dilution factor	Ionization solvent
0.5 mL of ACN (0.002 % FA and 0.1 mg L^{-1} IS)	0.5 mL of $CHCl_3$ and 0.02 mL of H_2O (both containing 0.002 % FA and 0.1 mg L^{-1} IS)	2	ACN:$CHCl_3$:H_2O 49:49:2 (0.002 % FA and 0.1 mg L^{-1} IS)
0.5 mL of ACN:$CHCl_3$:H_2O 49:49:2 (0.002 % FA and 0.1 mg L^{-1} IS)	0.5 mL of ACN:$CHCl_3$:H_2O 49:49:2 (0.002 % FA and 0.1 mg L^{-1} IS)		
0.5 mL of $CHCl_3$ (0.002 % FA and 0.1 mg L^{-1} IS)	0.5 mL of ACN and 0.02 mL of H_2O (both containing 0.002 % FA and 0.1 mg L^{-1} IS)		

Table S-3 List of selected chemical formulas that have been found with high (++) intensity (> 100 000 a.u.), low (+) intensity (> 1 000 a.u.) or that have barely or not (–) been found (< 1 000 a.u.) in *Brassica napus* and *Arabidopsis thaliana* (Col-0). Random artificial molecules are listed at the bottom of the table. The detected absence of these unexpected molecules supports the validity of the approach used for the identification of the chemical formulas. Cotyledons were extracted for 60 seconds with ACN/water 90:10 with 0.1 % formic acid and analysed with HRMS in negative ion mode. Fatty acids showed low ionization efficiencies under these conditions and are therefore not listed.

Molecular formula (neutral)	Theoretical mass $[M-H]^-$	Class	Tentative assignment	*B. napus*	*A. thaliana*	MS^2 fragments
$C_6H_{13}NO_2$	130.08735	Amino acid	(Iso)leucine	+	+	
$C_3H_7NO_2$	88.04040	Amino acid	Alanine	+	+	
$C_6H_{14}N_4O_2$	173.10440	Amino acid	Arginine	-	-	
$C_4H_8N_2O_3$	131.04622	Amino acid	Asparagine	+	+	
$C_4H_7NO_4$	132.03023	Amino acid	Aspartic acid	+	+	
$C_3H_7NSO_2$	120.01247	Amino acid	Cysteine	-	-	
$C_5H_9NO_4$	146.04588	Amino acid	Glutamic acid	+	+	
$C_5H_{10}N_2O_3$	145.06187	Amino acid	Glutamine	++	++	127.05<br>109.04
$C_6H_9N_3O_2$	154.06220	Amino acid	Histidine	-	-	
$C_6H_{14}N_2O_2$	145.09825	Amino acid	Lysine	-	-	
$C_5H_{11}NSO_2$	148.04377	Amino acid	Methionine	-	-	
$C_9H_{11}NO_2$	164.07170	Amino acid	Phenylalanine	-	-	
$C_5H_9NO_2$	114.05605	Amino acid	Proline	+	+	
$C_3H_7NO_3$	104.03532	Amino acid	Serine	+	+	
$C_4H_9NO_3$	118.05097	Amino acid	Threonine	+	+	
$C_{11}H_{12}N_2O_2$	203.08260	Amino acid	Tryptophan	-	-	
$C_9H_{11}NO_3$	180.06662	Amino acid	Tyrosine	-	-	
$C_5H_{11}NO_2$	116.07170	Amino acid	Valine	+	+	
$C_{10}H_{12}O_4$	195.06628	Aromatic	Acetosyringone	-	-	
$C_{12}H_{16}O_9$	303.07216	Aromatic	Benzentetrol-glucose	-	-	
$C_7H_6O_2$	121.02950	Aromatic	Benzoic acid	-	-	

$C_9H_8O_4$	179.03498	Aromatic	Caffeic acid	-	-	
$C_{15}H_{18}O_9$	341.08781	Aromatic	Caffeoyl-glucose	-	-	
$C_{13}H_{12}O_8$	295.04594	Aromatic	Caffeoyl-malate	-	-	
$C_{16}H_{18}O_9$	353.08781	Aromatic	Caffeoyl-quinate	-	-	
$C_{16}H_{16}O_8$	335.07724	Aromatic	Caffeoyl-shikimate	-	-	
$C_{13}H_{14}O_8$	297.06159	Aromatic	Caffeoyl-threonate	-	-	
$C_{10}H_{12}O_3$	179.07137	Aromatic	Canolol	-	-	
$C_6H_6O_2$	109.02950	Aromatic	Catechol	-	-	
$C_9H_8O_2$	147.04515	Aromatic	Cinnamic acid	-	-	
$C_{15}H_{18}O_7$	309.09798	Aromatic	Cinnamoyl-glucose	-	-	
$C_{13}H_{12}O_6$	263.05611	Aromatic	Cinnamoyl-malate	-	-	
$C_{16}H_{18}O_7$	321.09798	Aromatic	Cinnamoyl-quinate	-	-	
$C_{16}H_{16}O_6$	303.08741	Aromatic	Cinnamoyl-shikimate	-	-	
$C_{13}H_{14}O_6$	265.07176	Aromatic	Cinnamoyl-threonate	-	-	
$C_{10}H_{12}O_3$	179.07137	Aromatic	Coniferyl alcohol	-	-	
$C_9H_8O_3$	163.04007	Aromatic	Coumaric acid	-	-	
$C_{15}H_{18}O_8$	325.09289	Aromatic	Coumaroyl-glucose	-	-	
$C_{13}H_{12}O_7$	279.05103	Aromatic	Coumaroyl-malate	-	-	
$C_{16}H_{18}O_8$	337.09289	Aromatic	Coumaroyl-quinate	-	-	
$C_{16}H_{16}O_7$	319.08233	Aromatic	Coumaroyl-shikimate	-	-	
$C_{13}H_{14}O_7$	281.06668	Aromatic	Coumaroyl-threonate	-	-	
$C_{11}H_{12}O_4$	207.06628	Aromatic	Dimethoxycinnamic acid	-	-	
$C_{17}H_{22}O_9$	369.11911	Aromatic	Dimethoxycinnamoyl-glucose	-	-	
$C_{15}H_{16}O_8$	323.07724	Aromatic	Dimethoxycinnamoyl-malate	-	-	
$C_{18}H_{22}O_9$	381.11911	Aromatic	Dimethoxycinnamoyl-quinate	-	-	
$C_{18}H_{20}O_8$	363.10854	Aromatic	Dimethoxycinnamoyl-shikimate	-	-	
$C_{15}H_{18}O_8$	325.09289	Aromatic	Dimethoxycinnamoyl-threonate	-	-	
$C_{10}H_{10}O_4$	193.05063	Aromatic	Ferulic acid	-	-	
$C_{16}H_{20}O_9$	355.10346	Aromatic	Feruloyl-glucose	-	+	
$C_{14}H_{14}O_8$	309.06159	Aromatic	Feruloyl-malate	+	-	
$C_{26}H_{40}O_6$	447.27521	Aromatic	Feruloyl-palmitic acid	-	-	
$C_{17}H_{20}O_9$	367.10346	Aromatic	Feruloyl-quinate	-	-	
$C_{17}H_{18}O_8$	349.09289	Aromatic	Feruloyl-shikimate	-	-	
$C_{14}H_{16}O_8$	311.07724	Aromatic	Feruloyl-threonate	-	-	
$C_7H_6O_5$	169.01425	Aromatic	Gallic acid	-	-	
$C_{10}H_{10}O_5$	209.04555	Aromatic	Hydroxyferulic acid	-	-	
$C_{16}H_{20}O_{10}$	371.09837	Aromatic	Hydroxyferuloyl-glucose	-	-	
$C_{14}H_{14}O_9$	325.05651	Aromatic	Hydroxyferuloyl-malate	-	-	
$C_{17}H_{20}O_{10}$	383.09837	Aromatic	Hydroxyferuloyl-quinate	-	-	
$C_{17}H_{18}O_9$	365.08781	Aromatic	Hydroxyferuloyl-shikimate	-	-	
$C_{14}H_{16}O_9$	327.07216	Aromatic	Hydroxyferuloyl-threonate	-	-	
$C_{10}H_{10}O_3$	177.05572	Aromatic	Methoxycinnamic acid	-	-	
$C_{16}H_{20}O_8$	339.10854	Aromatic	Methoxycinnamoyl-glucose	-	-	
$C_{14}H_{14}O_7$	293.06668	Aromatic	Methoxycinnamoyl-malate	-	-	
$C_{17}H_{20}O_8$	351.10854	Aromatic	Methoxycinnamoyl-quinate	-	-	
$C_{17}H_{18}O_7$	333.09798	Aromatic	Methoxycinnamoyl-shikimate	-	-	
$C_{14}H_{16}O_7$	295.08233	Aromatic	Methoxycinnamoyl-threonate	-	-	
$C_6H_6O_3$	125.02442	Aromatic	Pyrogallol	-	-	
$C_{18}H_{16}O_8$	359.07724	Aromatic	Rosmarinic acid	-	-	
$C_{13}H_{16}O_8$	299.07724	Aromatic	Salicyl-glucose	-	-	
$C_{11}H_{12}O_4$	207.06628	Aromatic	Sinapaldehyde	-	-	
$C_{11}H_{12}O_5$	223.06120	Aromatic	Sinapinic acid	++	++	164.05<br>208.04<br>149.02
$C_{17}H_{22}O_{10}$	385.11402	Aromatic	Sinapoyl-glucose	+	++	223.06<br>205.05<br>190.02
$C_{15}H_{16}O_9$	339.07216	Aromatic	Sinapoyl-malate	++	++	223.06<br>133.01<br>183.01
$C_{27}H_{42}O_7$	477.28578	Aromatic	Sinapoyl-palmitic acid	-	-	
$C_{18}H_{22}O_{10}$	397.11402	Aromatic	Sinapoyl-quinate	-	-	
$C_{18}H_{20}O_9$	379.10346	Aromatic	Sinapoyl-shikimate	-	-	
$C_{15}H_{18}O_9$	341.08781	Aromatic	Sinapoyl-threonate	-	-	
$C_{11}H_{14}O_4$	209.08193	Aromatic	Sinapyl alcohol	-	-	
$C_9H_{10}O_4$	181.05063	Aromatic	Syringaldehyde	-	-	
$C_9H_{10}O_5$	197.04555	Aromatic	Syringic acid	-	-	
$C_8H_{10}O_3$	153.05572	Aromatic	Syringol	-	-	
$C_{13}H_{18}O_8$	301.09289	Aromatic	Tachioside	-	-	

$C_9H_8O_5$	195.02990	Aromatic	Trihydroxycinnamic acid	-	-	
$C_{15}H_{18}O_{10}$	357.08272	Aromatic	Trihydroxycinnamoyl-glucose	-	-	
$C_{13}H_{12}O_9$	311.04086	Aromatic	Trihydroxycinnamoyl-malate	-	-	
$C_{16}H_{18}O_{10}$	369.08272	Aromatic	Trihydroxycinnamoyl-quinate	-	-	
$C_{16}H_{16}O_9$	351.07216	Aromatic	Trihydroxycinnamoyl-shikimate	-	-	
$C_{13}H_{14}O_9$	313.05651	Aromatic	Trihydroxycinnamoyl-threonate	-	-	
$C_{12}H_{14}O_5$	237.07685	Aromatic	Trimethoxycinnamic acid	-	-	
$C_{18}H_{24}O_{10}$	399.12967	Aromatic	Trimethoxycinnamoyl-glucose	-	-	
$C_{16}H_{18}O_9$	353.08781	Aromatic	Trimethoxycinnamoyl-malate	-	-	
$C_{19}H_{24}O_{10}$	411.12967	Aromatic	Trimethoxycinnamoyl-quinate	-	-	
$C_{19}H_{22}O_9$	393.11911	Aromatic	Trimethoxycinnamoyl-shikimate	-	-	
$C_{16}H_{20}O_9$	355.10346	Aromatic	Trimethoxycinnamoyl-threonate	-	-	
$C_8H_8O_4$	167.03498	Aromatic	Vanillic acid	-	-	
$C_8H_8O_3$	151.04007	Aromatic	Vanillin	-	-	
$C_{14}H_{18}O_9$	329.08781	Aromatic	Vanilloyl-glucose	-	-	
$C_9H_{10}O_5$	197.04555	Aromatic	α-Hydroxyhydrocaffeic acid	-	-	
$C_5H_{10}O_4$	133.05063	Sugar	Deoxyribose	+	+	
$C_{12}H_{22}O_{11}$	341.10894	Sugar	Dihexose	+	+	
$C_6H_{12}O_6$	179.05611	Sugar	Hexose	++	++	
$C_5H_{10}O_5$	149.04555	Sugar	Pentose	+	+	
$C_{15}H_{12}O_8$	319.04594	Flavonoid	Dihydrogossypetin	-	-	
$C_{15}H_{10}O_8$	317.03029	Flavonoid	Gossypetin	-	-	
$C_{21}H_{20}O_{13}$	479.08311	Flavonoid	Gossypin	-	-	
$C_{15}H_{10}O_6$	285.04046	Flavonoid	Kaempferol	-	-	
$C_{21}H_{20}O_{12}$	463.08820	Flavonoid	Myricitrin	-	-	
$C_{15}H_{12}O_5$	271.06120	Flavonoid	Naringenin	-	-	
$C_{27}H_{32}O_{14}$	579.17193	Flavonoid	Naringin	-	-	
$C_{15}H_{10}O_7$	301.03538	Flavonoid	Quercetin	-	-	
$C_{15}H_{12}O_7$	303.05103	Flavonoid	Taxifolin	-	-	
$C_5H_5N_5$	134.04722	Nucleic acid	Adenine	-	-	
$C_4H_5N_3O$	110.03599	Nucleic acid	Cytosine	-	-	
$C_5H_5N_5O$	150.04213	Nucleic acid	Guanine	-	-	
$C_5H_6N_2O_2$	125.03565	Nucleic acid	Thymine	-	-	
$C_4H_4N_2O_2$	111.02000	Nucleic acid	Uracil	-	-	
$C_6H_8O_6$	175.02481	Others	Ascorbic acid	++	++	115.00<br>87.01<br>113.02
$C_6H_8O_7$	191.01973	Others	Citric acid	++	++	111.01<br>173.01<br>87.01
$C_6H_6O_6$	173.00916	Others	Dehydroascorbic acid	+	+	
$C_4H_4O_4$	115.00368	Others	Fumaric acid	+	+	
$C_7H_{14}O_8$	225.06159	Others	Glucoheptonic acid	+	+	
$C_6H_{12}O_7$	195.05103	Others	Gluconic acid	+	+	
$C_{10}H_{17}N_3SO_6$	306.07653	Others	Glutathione	+	+	
$C_3H_6O_4$	105.01933	Others	Glyceric acid	+	+	
$C_3H_8O_3$	91.04007	Others	Glycerol	-	-	
$C_5H_6O_5$	145.01425	Others	Ketoglutaric acid	+	+	
$C_4H_4O_4$	115.00368	Others	Maleic acid	+	+	
$C_4H_6O_5$	133.01425	Others	Malic acid	++	++	115.00<br>71.01<br>87.01
$C_3H_4O_4$	103.00368	Others	Malonic acid or<br>3-hydroxyperuvic acid	+	+	59.01
$C_5H_{10}O_4$	133.05063	Others	Monoacetylglycerol	-	+	
$C_{11}H_{19}NS_2O_{10}$	388.03776	Others	Progoitrin	++	-	
$C_3H_4O_3$	87.00877	Others	Pyruvic acid	+	+	
$C_7H_{12}O_6$	191.05611	Others	Quinic acid	-	-	
$C_7H_{10}O_5$	173.04555	Others	Shikimic acid	-	-	
$C_4H_6O_6$	149.00916	Others	Tartaric acid	-	-	
$C_4H_8O_5$	135.02990	Others	Threonic acid	+	+	
$C_7H_5NS_2$	165.97906	Artificial	2-Mercaptobenzothiazol	-	-	
$C_{14}H_{22}N_2O_3$	265.15577	Artificial	Atenolol	-	-	
C_7H_5NSO	150.00191	Artificial	Benzisothiazolinone	-	-	
$C_{15}H_{16}O_2$	227.10775	Artificial	Bisphenol A	-	-	
$C_{13}H_{12}O_2$	199.07645	Artificial	Bisphenol F	-	-	
$C_{15}H_{12}N_2O$	235.08769	Artificial	Carbamazepine	-	-	
$C_9H_9N_3O_2$	190.06220	Artificial	Carbendazim	-	-	
$C_{17}H_{21}NO_4$	303.14761	Artificial	Cocaine	-	-	

$C_{12}H_{17}NO$	190.12374	Artificial	DEET	-	-	
$C_{16}H_{22}O_4$	277.14453	Artificial	Dibutyl phthalate	-	-	
$C_{21}H_{23}NO_5$	368.15035	Artificial	Heroin	-	-	
$C_{20}H_{25}N_3O$	322.19249	Artificial	LSD	-	-	
$C_{11}H_{15}NO_2$	192.10300	Artificial	MDMA	-	-	
$C_4H_{11}N_5$	128.09417	Artificial	Metformin	-	-	
C_4H_5NSO	114.00191	Artificial	Methylisothiazolinon	-	-	
$C_6H_9N_3O_3$	170.05712	Artificial	Metronidazole	-	-	
C_5H_5NSO	126.00191	Artificial	Pyrithione	-	-	
$C_7H_5NSO_3$	181.99174	Artificial	Saccharin	-	-	
$C_{10}H_{11}N_3SO_3$	252.04484	Artificial	Sulfamethoxazole	-	-	
$C_{13}H_{10}N_2O_4$	257.05678	Artificial	Thalidomide	-	-	
$C_8H_{13}N_3SO_4$	246.05540	Artificial	Tinidazole	-	-	
$C_9H_6N_2O_2$	173.03565	Artificial	Toluene diisocyanate	-	-	
C_7H_9N	106.06622	Artificial	Toluidine	-	-	
$C_7H_5N_3O_6$	226.01055	Artificial	Trinitrotoluene	-	-	
$C_{24}H_{29}N_5O_3$	434.21976	Artificial	Valsartan	-	-	

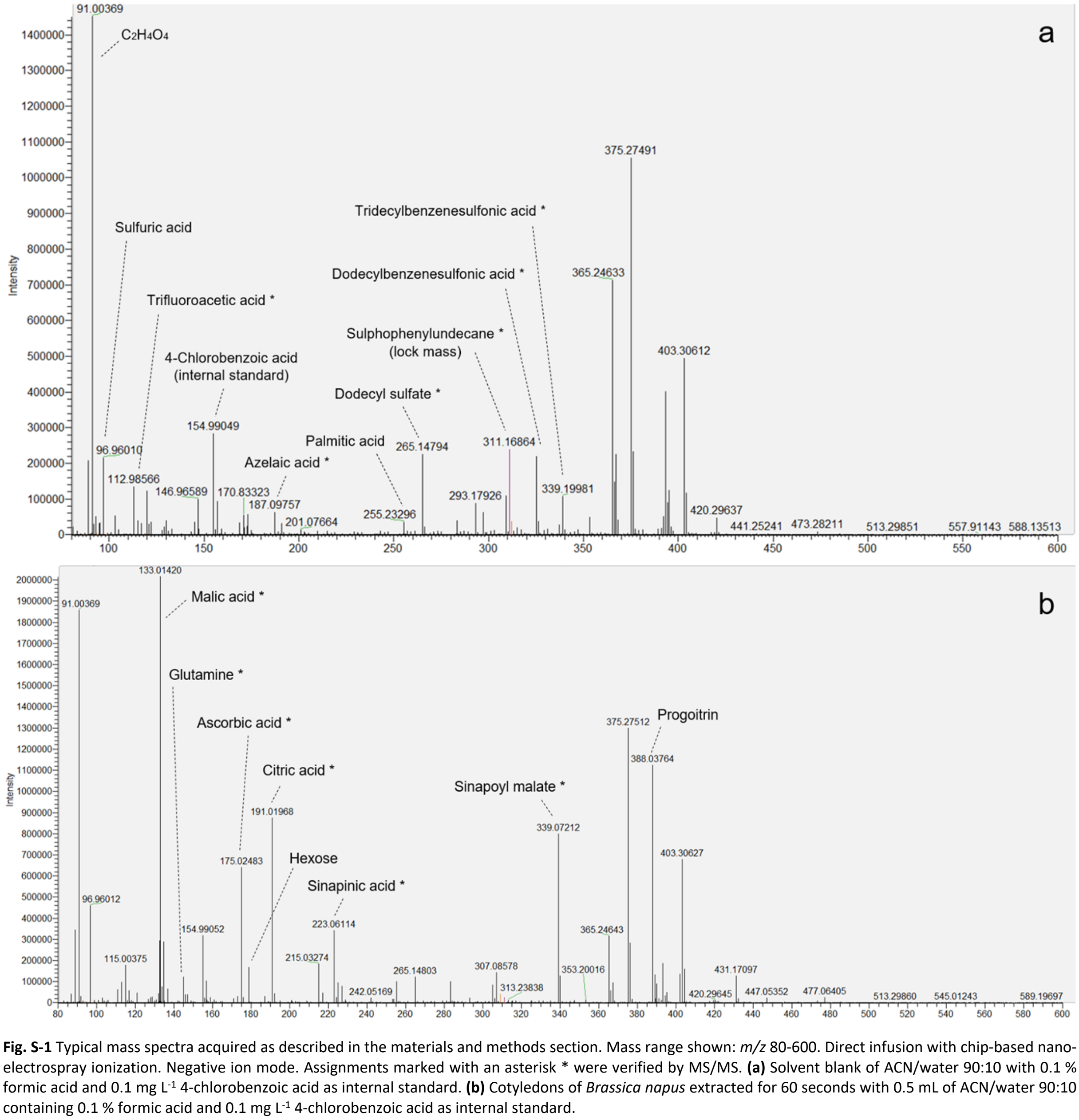

Fig. S-1 Typical mass spectra acquired as described in the materials and methods section. Mass range shown: *m/z* 80-600. Direct infusion with chip-based nano-electrospray ionization. Negative ion mode. Assignments marked with an asterisk * were verified by MS/MS. **(a)** Solvent blank of ACN/water 90:10 with 0.1 % formic acid and 0.1 mg L^{-1} 4-chlorobenzoic acid as internal standard. **(b)** Cotyledons of *Brassica napus* extracted for 60 seconds with 0.5 mL of ACN/water 90:10 containing 0.1 % formic acid and 0.1 mg L^{-1} 4-chlorobenzoic acid as internal standard.

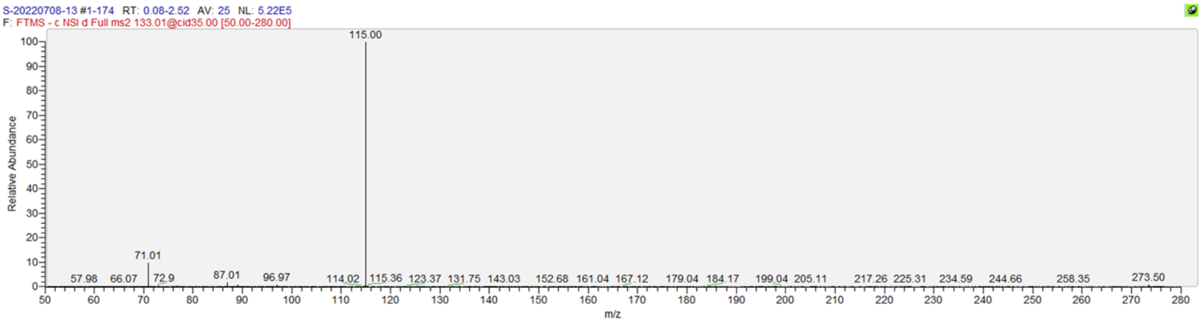

Fig. S-2 MS^2 spectrum of the signal at *m/z* 133.01 (malic acid). Fragmentation was done with direct infusion in negative mode (ACN:H_2O 90:10, 0.1 % FA).

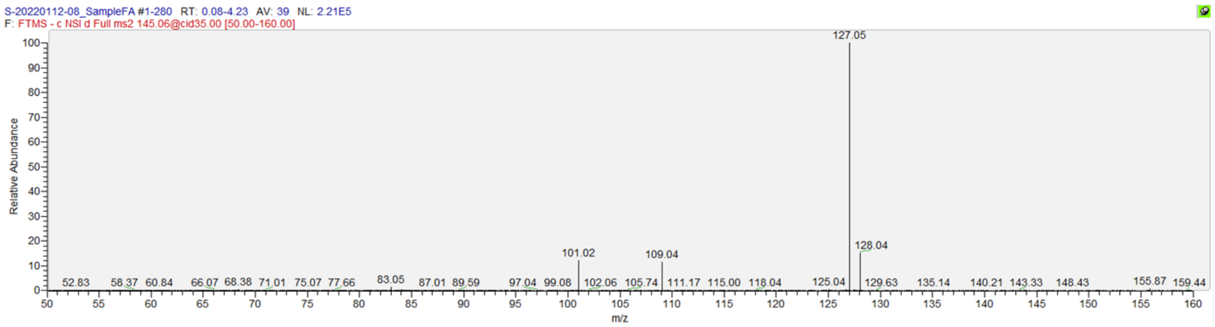

Fig. S-3 MS^2 spectrum of the signal at *m/z* 145.06 (glutamine). Fragmentation was done with direct infusion in negative mode (ACN:H_2O 90:10, 0.1 % FA).

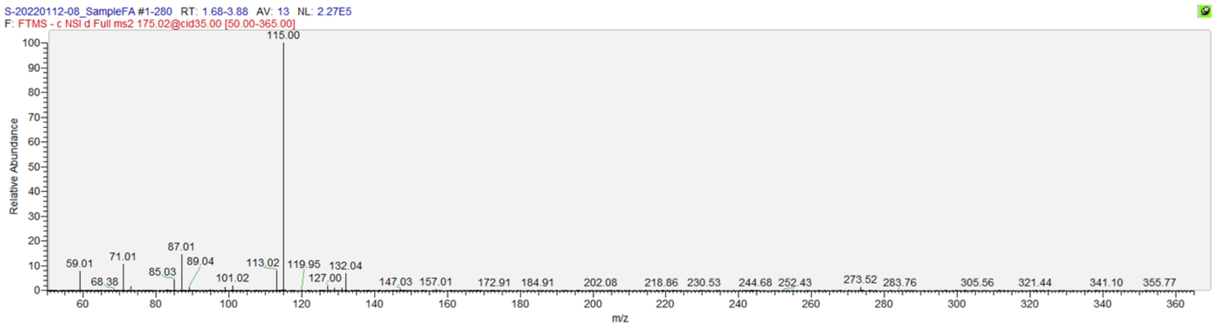

Fig. S-4 MS^2 spectrum of the signal at *m/z* 175.02 (ascorbic acid). Fragmentation was done with direct infusion in negative mode (ACN:H_2O 90:10, 0.1 % FA).

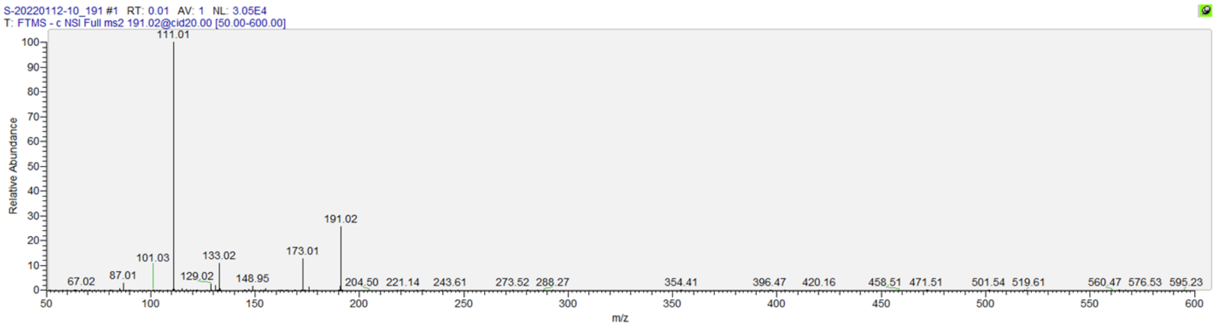

Fig. S-5 MS^2 spectrum of the signal at *m/z* 191.02 (citric acid). Fragmentation was done with direct infusion in negative mode (ACN:H_2O 90:10, 0.1 % FA).

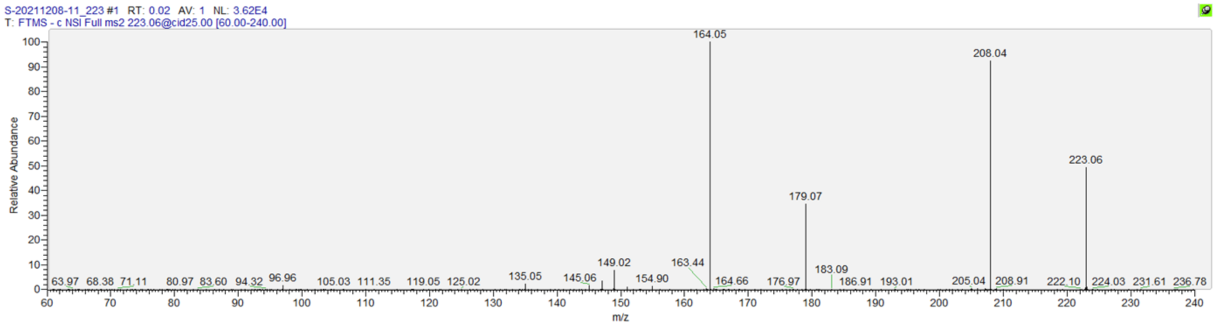

Fig. S-6 MS^2 spectrum of the signal at *m/z* 223.06 (sinapinic acid). Fragmentation was done with direct infusion in negative mode (ACN:H_2O 90:10, 0.1 % FA).

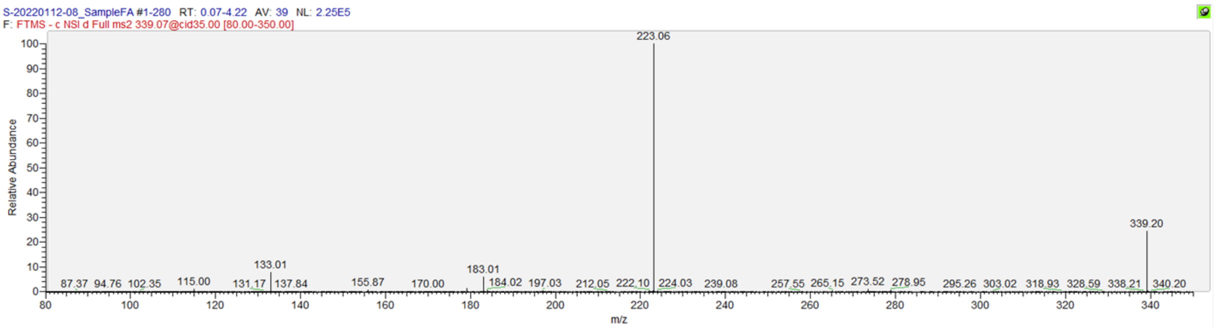

Fig. S-7 MS^2 spectrum of the signal at *m/z* 339.07 (sinapoyl malate). Fragmentation was done with direct infusion in negative mode (ACN:H_2O 90:10, 0.1 % FA).

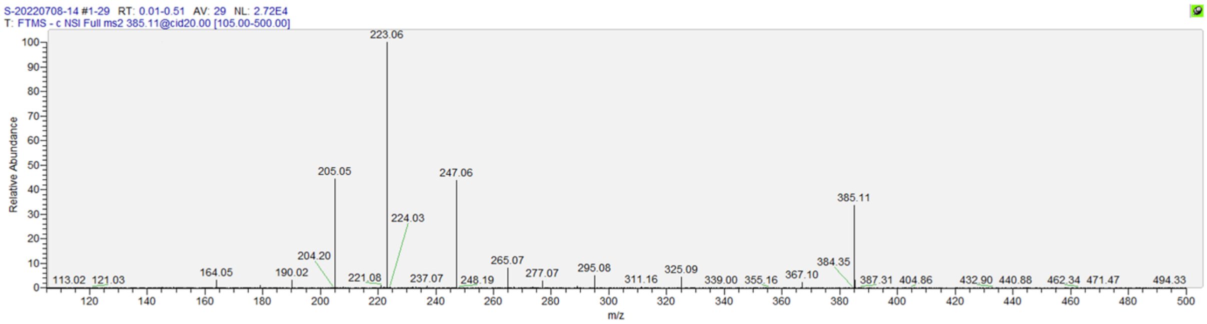

Fig. S-8 MS^2 spectrum of the signal at *m/z* 385.11 (sinapoyl glucose). Fragmentation was done with direct infusion in negative mode (ACN:H_2O 90:10, 0.1 % FA).
